# Killing of *Staphylococcus aureus* and *Salmonella enteritidis* and neutralization of lipopolysaccharide by 17-residue bovine lactoferricins: improved activity of Trp/Ala-containing molecules

**DOI:** 10.1038/srep44278

**Published:** 2017-03-13

**Authors:** Ya Hao, Na Yang, Xiumin Wang, Da Teng, Ruoyu Mao, Xiao Wang, Zhanzhan Li, Jianhua Wang

**Affiliations:** 1 Key Laboratory of Feed Biotechnology, Ministry of Agriculture, Beijing 100081, China; 2 Gene Engineering Laboratory, Feed Research Institute, Chinese Academy of Agricultural Sciences, Beijing 100081, China

## Abstract

Bovine lactoferricin (LfcinB) has potent antibacterial, antifungal and antiparasitic activities but is also hemolytic. Our objective was to identify LfcinB17-31 derivatives with reduced hemolysis and improved antimicrobial activity via substituting Cys3, Arg4, Gln7, Met10, and Gly14 with more hydrophobic residues. Two peptides, Lfcin4 and Lfcin5, showed higher activity against *Staphylococcus aureus* and *Salmonella enteritidis* and lower hemolytic activity than the parent peptide LfcinB17-31. These peptides permeabilized the outer and inner membranes of *S. enteritidis*; however, Lfcin5 did not permeabilize the inner membrane of *S. aureus*. Gel retardation and circular dichroism spectra showed that Lfcin4 and Lfcin5 bound to bacterial genomic DNA. Lfcin4 inhibited DNA, RNA and protein synthesis. Both peptides induced the peeling of membranes and the lysis of *S. enteritidis*. At doses of 10 and 15 mg/kg, Lfcin4 and Lfcin5 reduced the bacterial counts in infected thigh muscles by 0.03‒0.10 and 0.05‒0.63 log_10_ CFU/g of tissue, respectively, within 10 h. Lfcin4 and Lfcin5 enhanced the survival rate of endotoxemic mice; reduced serum IL-6, IL-1β and TNF-α levels; and protected mice from lipopolysaccharide-induced lung injury. These data suggest that Lfcin4 and Lfcin5 may be antimicrobial and anti-endotoxin peptides that could serve as the basis for the development of dual-function agents.

*Staphylococcus aureus* and *Salmonella enteritidis* are leading causes of infection at numerous sites in the body (including skin, soft tissue, blood, and lungs) in both humans and animals^1^,^2^,^3^. The use of antibiotics is critical for the treatment of these infectious bacterial diseases. However, multidrug-resistant isolates of *S. aureus* and *S. enteritidis* have been present for many years^4^. Thus, it is of critical importance to identify alternatives to traditional antibiotics. Additionally, the endotoxin lipopolysaccharide (LPS) found on cell membranes of Gram-negative bacteria, a primary trigger of sepsis, can stimulate host cells to produce a large amount of proinflammatory cytokines that play a pivotal role in the pathogenesis of multiple organ failure^5^,^6^. Currently, the neutralization of LPS is one of the therapeutic strategies being investigated for sepsis; however, no therapeutic agents thus far have been efficacious enough to protect the host from tissue damage and organ failure induced by LPS. Thus, there is a pressing need for new effective agents that can neutralize LPS^7^.

The bovine lactoferricin (LfcinB) peptide is generated by the gastric pepsin cleavage of the N-terminal region of the iron-binding protein lactoferrin. LfcinB consists of 25 amino acids, corresponding to residues 17–41 in lactoferrin, and is a bioactive peptide that possesses antibacterial, antifungal, antiviral, antiparasitic, antitumor, and immunological activities^8^,^9^,^10^,^11^,^12^,^13^,^14^,^15^,^16^,^17^. A shorter version of LfcinB containing amino acids 17 to 31 (LfcinB17–31) was found to have slightly less antimicrobial activity against *S. aureus* and *Escherichia coli* than LfcinB17-41^18^.

A previous study showed that the core hexapeptide (RRWQWR) of LFcinB can cause membrane disturbances in model membranes and penetrate HeLa cells^19^. Additionally, the Trp residues at positions 6 and 8 of LfcinB17-31 are crucial for its high antibacterial activity^20^. The largest loss of antimicrobial activity is found when either of these Trp residues is replaced by Ala. A single Ala substitution at Cys3, Gln7 or Gly14 results in an approximately 1-fold increase in the antibacterial activity of LfcinB17-31 against *E. coli*, and other Ala substitutions display reduced antibacterial activity against *S. aureus*^20^. Substitutions of Cys3, Gln7 and Ala14 with Trp have revealed that the antimicrobial activity increases with the amount of Trp residues, especially against *S. aureus*^21^. The aromatic Trp residues are hypothesized to function as an anchor in membrane proteins and act as a needle that pulls the α-helix across phospholipid membranes^21^. Additionally, the Arg residues were reported to be critical for antimicrobial activity against *Mycobacterium avium*^22^. All of the Arg substitutions (at Lys2, Lys11 and Lys12) were slightly more active than the parent peptide. Conversely, the substitution of Lys at Arg4, Arg5 and Arg9 resulted in significantly less antimicrobial activity than the corresponding original peptide^22^.

Some antimicrobial peptides (AMPs) exhibit remarkable specificity for particular bacterial species. LfcinB can damage the outer membrane of bacteria by interaction with LPS or teichoic acid, and it is hypothesized that the main function of LfcinB is to increase the permeability of the cytoplasmic membrane of both Gram-positive and Gram-negative bacteria, a mechanism closely resembling that of the class L antibiotic peptides^23^,^24^. LfcinB inhibits DNA synthesis in *E. coli* UC 6782 and interferes with DNA, RNA and protein synthesis in *Bacillus subtilis*^25^. It was determined that the mode of action of LfcinB in *B. subtilis* may differ from that in *E. coli* and that LfcinB may induce an SOS-like response in *E. coli*^25^. Ho *et al*. identified the intracellular targets of LfcinB using *E. coli* K12 proteome microarrays and found that LfcinB binds to two response regulators, BasR and CreB, members of the two-component system family. They further showed that LfcinB inhibited the growth of bacteria by directly influencing the phosphorylation of a two-component system protein^26^. However, the exact mode of action of LfcinB or its derivatives against *S. aureus* and *S. enteritidis* has not yet been elucidated.

In the present study, on the basis of previous reports wherein some residues of Phe1, Lys2, Trp6, Trp8, Arg9, Lys11, Lys12, and Ala15 are found to be essential for antibacterial activity^18^,^19^,^20^,^22^,^27^, we performed a systematic analysis of residues in the 3^rd^, 4^th^, 7^th^, 10^th^, and 14^th^ positions of LfcinB17–31 by substituting the hydrophobic amino acids (Ala, Phe and Trp) at each position. LfcinB17-31 and its derivatives were synthesized and their antimicrobial activities against Gram-positive and Gram-negative bacteria were evaluated. One possible mechanism of action of LfcinB derivatives against *S. aureus* and *S. enteritidis* was further elucidated. Additionally, the *in vivo* antibacterial activities of LfcinB derivatives were examined in a mouse thigh model of *S. aureu*s infection, as well as their endotoxin-neutralizing activities in C57BL/6 mice challenged with LPS.

## Results

### Physicochemical parameters, structures and electrostatic potentials on the surface of LfcinB17-31 and its derivatives

The amino acid sequences and key physicochemical parameters of the LfcinB17-31 derivatives are summarized in Table 1. The physicochemical properties of Lfcin2 were almost equal to those of the parent peptide, Lfcin1. The net charge of peptides Lfcin3-Lfcin6 was lower than that of Lfcin1. The Lfcin3, Lfcin4 and Lfcin5 peptides had the highest α-helical content at 64.7% and Lfcin6 had the lowest α-helix content at 35.2%. Lfcin2 contained a lower helical content (52.9%) compared to Lfcin1. The hydrophobicity of Lfcin3-Lfcin6 increased from −1.207 to −0.376, indicating that they are more hydrophobic than Lfcin1 and Lfcin2; this is likely due to the addition of a methyl group from the Gly/Cys/Gln to Ala residue substitution^28^. The instability index of Lfcin2-Lfcin6 ranged from 28.82 to 46.67. The Boman index, which is a measure of peptide affinity to proteins and the ability to establish bio-interactions, of LfcinB17-31 derivatives was in the range of 1.45‒3.02 kcal/mol.

The two-dimensional (2D) and three-dimensional (3D) structure modeling of Lfcin3, Lfcin4 and Lfcin5 reveals similarities to their parent peptide, which adopts a typical α-helix (Supplementary Figs S1A and S1B). However, Lfcin4 and Lfcin5 appeared to have a more irregular coil in the C-terminus (35.3%), whereas Lfcin6 lost its α-helical structure and formed a turn (70.6%) and an irregular coil (29.4%) (Supplementary Table S1), perhaps due to the replacement of Arg4 and Met10 by Trp. The electrostatic potentials of Lfcin3, Lfcin4 and Lfcin5 (from ±84.244 to ±85.553 kT/e) were lower than those of Lfcin1 (±98.263 kT/e), Lfcin2 (±93.267 kT/e) and Lfcin6 (±86.594 kT/e) (Supplementary Fig. S1C), revealing their distinct cationic and amphipathic characters. This difference likely results in different permeabilization abilities of these peptides through electrostatic and hydrophobic interactions with bacterial membranes^29^.

### Lfcin4 and Lfcin5 display antibacterial activity with low hemolytic activity

**Figure 1:**
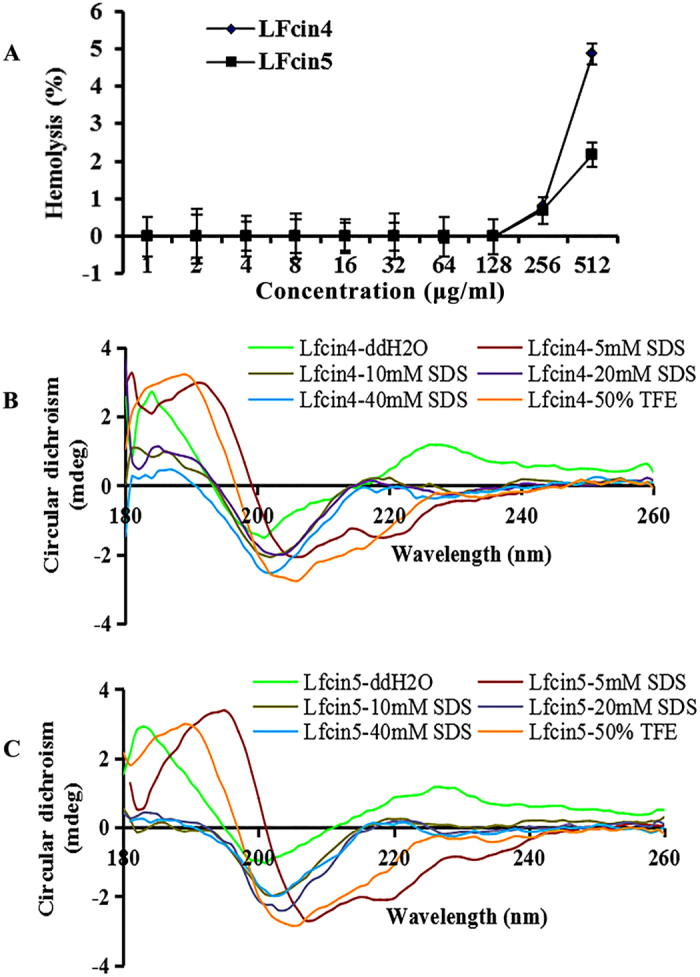
Hemolysis and CD spectra of the secondary structures of Lfcin4 and Lfcin5. (**A**) Hemolytic activity of Lfcin4 and Lfcin5 against mouse erythrocytes. The results are presented as the mean ± SEM (n = 3). **(B,C)** CD spectra of the secondary structures of Lfcin4 **(B)** and Lfcin5 **(C)**.

As shown in Table 2, Lfcin4 and Lfcin5 showed higher activity against *E. coli, S. enteritidis* and *S. aureus* than against Lfcin1. The MIC values for Lfcin4 and Lfcin5 against *S. enteritidis* CVCC3377, *S. aureus* ATCC25923 and *S. aureus* ATCC43300 were in the range of 2 to 64 μg/ml. Lfcin6 exhibited the lowest antimicrobial activity against the test strains. Similar to Lfcin1, higher MICs (>128 μg/ml) for Lfcin2 to Lfcin6 against *S. pullorum* CVCC1789 and CVCC1802, and *P. aeruginosa* CICC10419 and CICC21630 were observed. Moreover, Lfcin4 and Lfcin5 displayed very low hemolytic activity against mouse erythrocytes (Fig. 1A). Even at a high concentration (512 μg/ml), the hemolytic activity of Lfcin4 and Lfcin5 was 4.87% and 2.2%, respectively, which is lower than that of the parent peptide, Lfcin1 (approximately 7.5% hemolysis at 256 μg/ml)^14^.

### Circular dichroism (CD) spectra of Lfcin4 and Lfcin5

Anionic surfactants such as sodium dodecyl sulfate (SDS) and fluorinated alcohols such as trifluoroethanol (TFE) are often used to mimic the cell membrane environment^30^. To analyze the structural features of Lfcin4 and Lfcin5, the CD spectra of peptides were measured in ddH_2_O, 5‒40 mM SDS and 50% TFE buffer, respectively. The secondary structures of Lfcin4 and Lfcin5 in ddH_2_O were characterized predominantly by antiparallel and random coils with a characteristic positive maximum at 230 nm and a negative minimum at 200 nm (Fig. 1B and C). The CD spectra of Lfcin4 and Lfcin5 showed an increase in α-helical content in SDS and TFE buffer (>11.8% α-helicity) (Supplementary Tables S2 and S3) relative to the parent peptide (<10% α-helicity)^20^. This result indicates that both SDS and TFE buffer are favorable for the formation of an α-helical structure.

### Lfcin4 and Lfcin5 affect the cell surface hydrophobicity of bacterial cells

**Figure 2:**
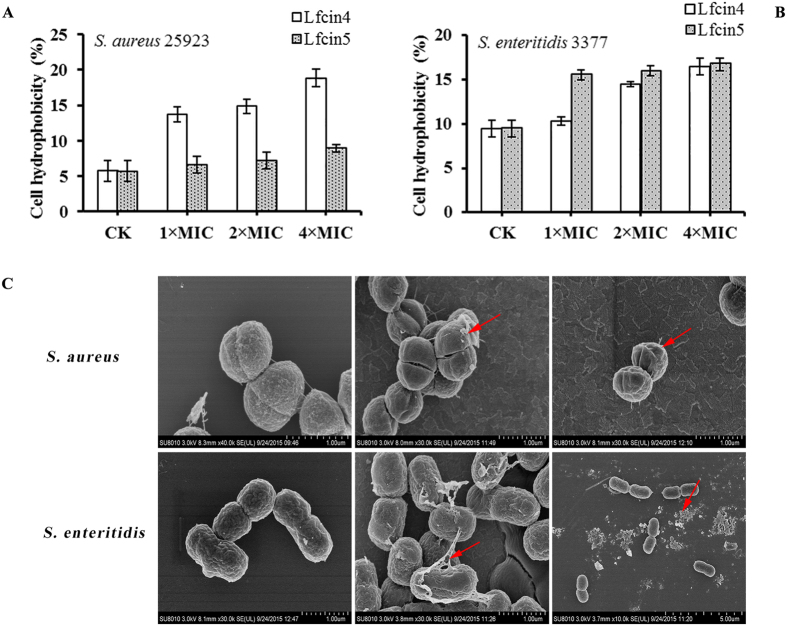
Effects of Lfcin4 and Lfcin5 on the cell surface hydrophobicity and cell morphology of bacteria. **(A,B)** Effects of Lfcin4 and Lfcin5 on the cell surface hydrophobicity of *S. aureus* ATCC25923 **(A)** and *S. enteritidis* CVCC3377. **(B)** The results are presented as the mean ± SEM (n = 3). **(C)** SEM photographs of *S. aureus* ATCC25923 and *S. enteritidis* CVCC3377 cells with or without 1 × MIC Lfcin4 and Lfcin5.

As shown in Fig. 2A and B, the cell surface hydrophobicity of *S. aureus* ATCC25923 and *S. enteritidis* CVCC3377 treated with peptides was positively correlated with the concentration of Lfcin4 and Lfcin5. For *S. aureus* ATCC25923, treatment with Lfcin4 resulted in an increased cell surface hydrophobicity from 5.27% to 18.87% (Fig. 2A), whereas Lfcin5 caused a relatively small change in cell surface hydrophobicity (from 5.27% to 8.95%). The cell surface hydrophobicity of *S. enteritidis* CVCC3377 increased from 9.43% to 16.45% and from 9.43% to 16.74% following treatment with Lfcin4 and Lfcin5, respectively, which is markedly higher than the untreated controls. Lfcin5 exhibited higher cell surface hydrophobicity than Lfcin4 (Fig. 2B). Changes in cell surface hydrophobicity are one of the noticeable effects of peptide interactions with bacterial cells, which may be correlated with structural alterations of bacterial cell surfaces^31^.

### Scanning electron microscopy (SEM) observations

Scanning electron microscopy (SEM) was used to directly observe the effects of 1 × MIC Lfcin4 or Lfcin5 treatment on the cell morphology and integrity of *S. aureus* ATCC25923 and *S. enteritidis* CVCC3377. As shown in Fig. 2C, untreated *S. aureus* cells displayed an intact cell morphology. After treatment with 1 × MIC Lfcin4 or Lfcin5 for 2 h, the *S. aureus* cells appeared smaller, suggesting that the cells shrunk or released intracellular components. In untreated control groups, a normal intact cell morphology was observed in *S. aureus*. For *S. enteritidis* CVCC3377, treatment with Lfcin4 or Lfcin5 induced membrane peeling in approximately 30% of cells and lysis in approximately 50% of cells.

### Lfcin4 and Lfcin5 permeabilize model and bacterial membranes

#### Permeabilization of model membranes

**Figure 3:**
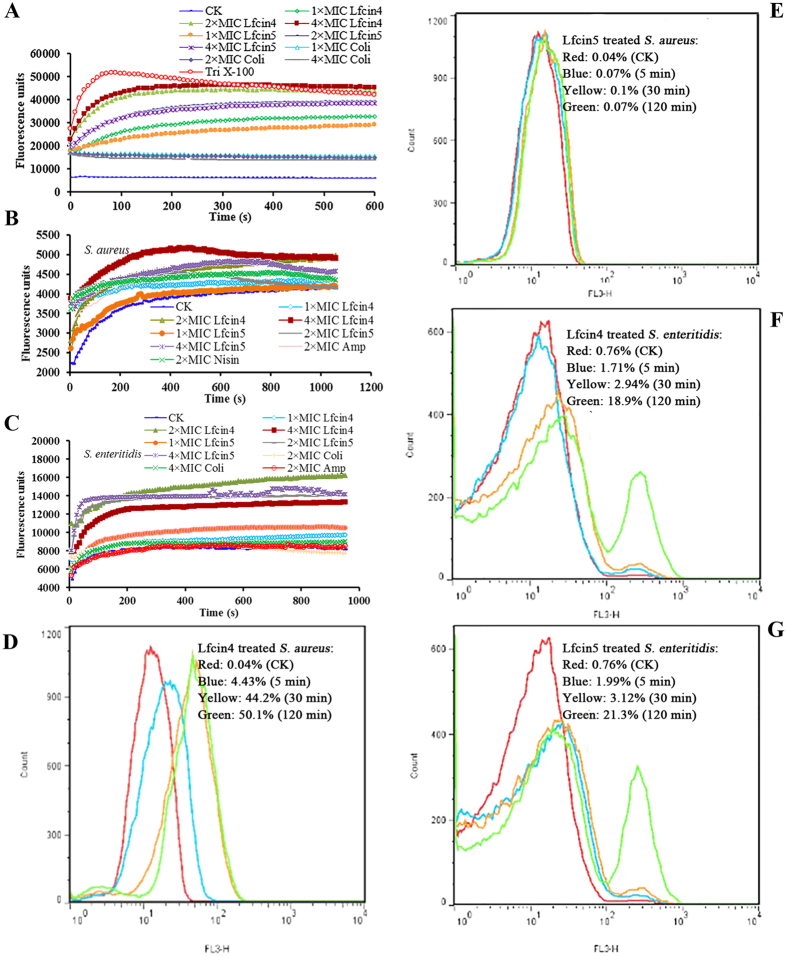
Effects of Lfcin4 and Lfcin5 on the biological membrane. **(A)** Leakage from POPC/POPG (1:3) vesicles induced by different concentrations of Lfcin4 and Lfcin5. Large unilamellar vesicles (LUVs) loaded with calcein were incubated with Lfcin4 and Lfcin5 and leakage of calcein was monitored for 10 min on a Tecan Infinite M200 PRO plate reader. **(B,C)** Time-response curve of the outer membrane permeabilization of *S. aureus* ATCC25923 **(B)** and *S. enteritidis* CVCC337 **(C)** cells treated with Lfcin4 and Lfcin5 in the presence of NPN. PBS treatment was used as a negative control. Treatment with ampicillin and colistin were used as positive controls. Amp: ampicillin; Coli: colistin. **(D–G)** Fluorescence-activated cell sorting (FACS) analysis of PI staining in *S. aureus* ATCC25923 **(D,E)** and *S. enteritidis* CVCC337 **(F,G)** cells treated with Lfcin4 **(D,F)** and Lfcin5 **(E,G)**.

To investigate whether Lfcin4 or Lfcin5 causes membrane disruption of bacterial cells, calcein was used as a marker for membrane damage. The release of calcein from liposomes was fluorometrically monitored following exposure to Lfcin4 or Lfcin5 peptides. As shown in Fig. 3A, both Lfcin4 and Lfcin5 induced the leakage of 2-oleoyl-1-pamlitoyl-sn-glyecro-3-phosphocholine/2-oleoyl-1-pamlitoyl-sn-glyecro-3-glycerol (POPC/POPG) vesicles. In *E. coli*, Lfcin4 treatment resulted in 45.5%, 90.6% and 98.4% dye leakage from vesicles at 5 min when used at 1×, 2× and 4× MIC, respectively. Treatment with Lfcin5 caused a similar result, with 30.1‒70.3% leakage within 5 min (data not shown). These results indicate that both Lfcin4 and Lfcin5 have potent ability to permeabilize the lipid bilayer in a time- and concentration-dependent manner and that Lfcin4 is more effective at permeabilizing bilayers than Lfcin5.

#### Permeabilization of the outer membrane in S. aureus and S. enteritidis

The hydrophobic fluorophoric probe N-phenyl-1-naphthylamine (NPN) is normally prevented from entering cells because it cannot penetrate the outer membrane. Once the outer membrane is damaged, NPN is partitioned into the interior of the outer membrane, thereby exhibiting strong fluorescence in this hydrophobic environment. As shown in Fig. 3B and C, both peptides instantly permeabilized the outer membrane of *S. aureus* ATCC25923 and *S. enteritidis* CVCC337 within 1 min, and higher concentrations of Lfcin4 and Lfcin5 resulted in a stronger NPN signal, indicating that the peptides made the outer membrane more permeable. These results suggest that both Lfcin4 and Lfcin5 induce a time- and concentration-dependent NPN fluorescence increase in intact bacterial cells and that the permeabilization capacity of Lfcin4 is higher than that of Lfcin5.

#### Permeabilization of the inner membrane in S. aureus and S. enteritidis

Propidium iodide (PI) is commonly used as a cell death marker because it is excluded by intact plasma membranes and can only enter cells that have lost their membrane integrity. The fluorescence of cells stained with PI was used as an indicator to evaluate cell membrane integrity and was measured by flow cytometry^32^. In the absence of peptides, the percentage of *S. aureus* ATCC25923 and *S. enteritidis* CVCC3377 cells stained with PI was 0.04% and 0.76%, respectively, indicating the presence of intact healthy cell membranes. The percentage of PI-positive *S. aureus* ATCC25923 cells treated with 1 × MIC Lfcin4 for 5 min, 30 min and 120 min was 4.30%, 44.20% and 50.10%, respectively (Fig. 3D); for *S. enteritidis* CVCC3377, Lfcin4 treatment resulted in 1.17%, 2.94% and 18.90% positive PI staining (Fig. 3F). A dose-dependent increase in PI fluorescence indicated that Lfcin4 damaged the cell membranes of *S. aureus* ATCC25923 and *S. enteritidis* CVCC3377. After treatment with 1 × MIC Lfcin5, the percentage of PI-positive *S. enteritidis* CVCC3377 cells was 1.99% (5 min), 3.12% (30 min) and 21.30% (120 min); however, Lfcin5 did not permeabilize the inner membrane of *S. aureus* ATCC25923 (Fig. 3E and G), in agreement with the SEM images.

### Lfcin4 and Lfcin5 bind to bacterial genomic DNA

**Figure 4:**
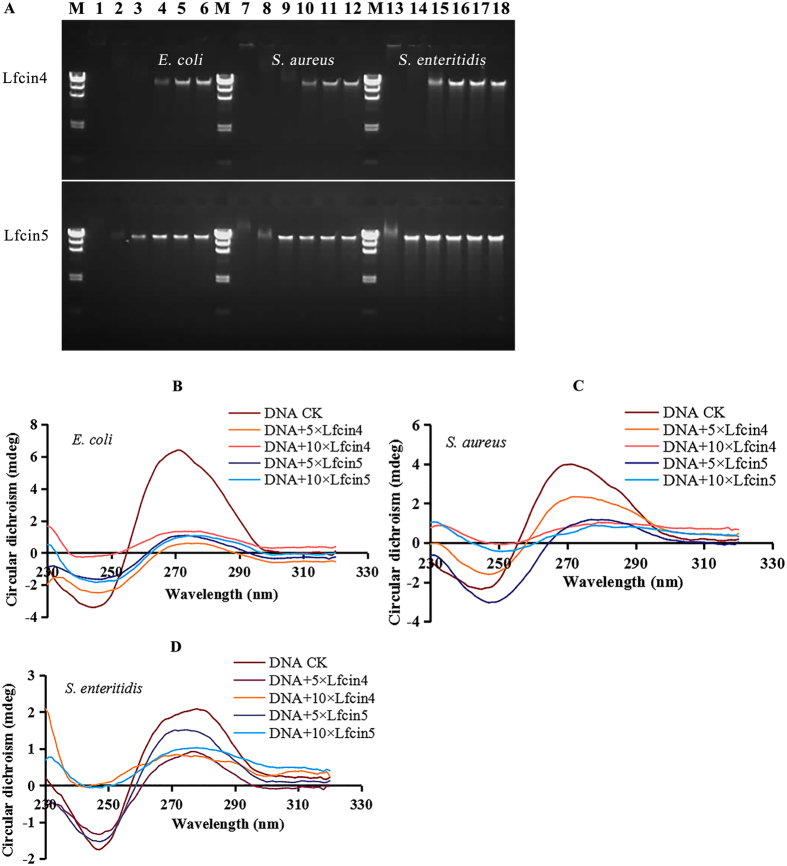
*In vitro* binding of Lfcin4 and Lfcin5 to bacterial genomic DNA. **(A)** Gel retardation analysis of the binding of Lfcin4 and Lfcin5 to genomic DNA. M: DNA marker λDNA/*Hind*III. Lanes 1–6: genomic DNA from *E. coli* CICC21530; Lanes 7–12: genomic DNA from *S. aureus* ATCC25923; Lanes 13–18: genomic DNA from *S. enteritidis* CVCC3377. The mass ratios of peptide to genomic DNA were 10, 5, 2.5, 1, 0.5, and 0. Full-length gels are presented in Supplementary Figure S2. **(B–D)** CD spectra of genomic DNA from *E. coli* CICC21530 **(B)**, *S. aureus* ATCC25923 **(C)** and *S. enteritidis* CVCC3377 **(D)** in the presence of Lfcin4 and Lfcin5. The mass ratios of peptide to DNA were 5 and 10.

An electrophoretic mobility shift assay (EMSA) was used to evaluate the binding affinity of each peptide to bacterial genomic DNA^33^. Lfcin4 and Lfcin5 interacted with the genomic DNA (Fig. 4A, Supplementary Fig. S2). Lfcin4 impaired the migration of the genomic DNA from *E. coli* O157 and *S. aureus* ATCC25923 at mass ratios over 1.0 (peptide:DNA) and from *S. enteritidis* CVCC3377 at a mass ratio greater than 2.5, demonstrating the intrinsic DNA-binding ability of Lfcin4. For Lfcin5, no DNA bands were observed on the gel for *E. coli* or *S. aureus* at mass ratios of 10; however, a fraction of the genomic DNA of *S. enteritidis* migrated into the gel. This finding suggests that DNA-binding activity of Lfcin4 is higher than that of Lfcin5. The DNA-binding properties of peptides were further supported by the following CD assay.

As shown in Fig. 4B,C and D, the DNA CD spectra were dramatically changed when peptides bound to DNA from *E. coli, S. aureus* and *S. enteritidis*, which contained a negative band at approximately 250 nm and a positive band at approximately 275 nm. There was a greater decrease in the CD amplitude but no obvious shift, suggesting that Lfcin4 and Lfcin5 interact with genomic DNA and alter the DNA conformation^34^.

### Lfcin4 inhibits the synthesis of DNA, RNA and protein precursors

**Figure 5:**
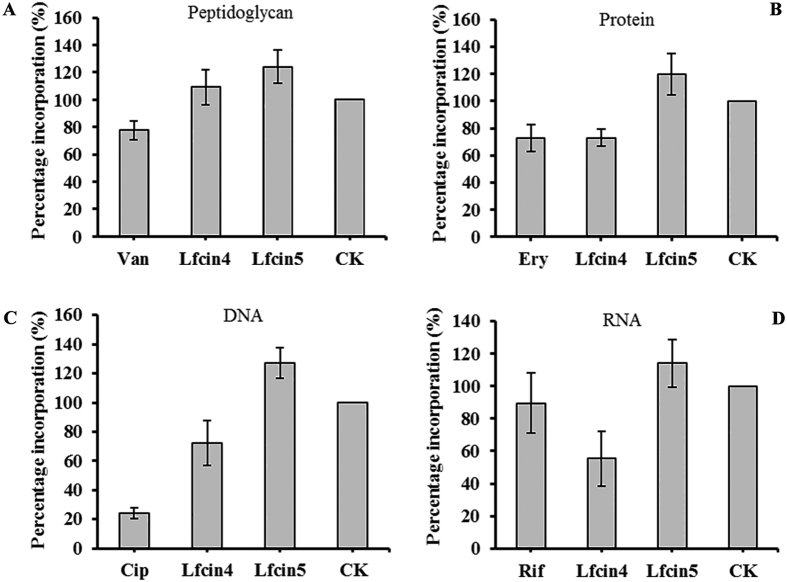
Effects of Lfcin4 and Lfcin5 on macromolecular synthesis in *S. aureus* ATCC25923. Incorporation of ^3^H-glucosamine hydrochloride (peptidoglycan) **(A)**, ^3^H-leucine (protein) **(B)**, ^3^H-thymidine (DNA) **(C)**, and ^3^H-uridine (RNA) **(D)** was determined in cells treated with 1 × MIC Lfcin4 and Lfcin5. Van: vancomycin (2 × MIC); Ery: erythromycin (2 × MIC); Cip: ciprofloxacin (8 × MIC); Rif: rifampicin (4 × MIC). Antibiotics were used as controls. Results are presented as the mean ± SEM (n = 3).

Despite the focus on bacterial membranes as targets for AMPs, several AMPs have additional intracellular targets^25^. The incorporation of radioactive precursors into DNA, RNA, peptidoglycans, and proteins was used to evaluate the effects of Lfcin4 and Lfcin5 on macromolecular synthesis in *S. aureus* ATCC25923 cells. Lfcin4 inhibited the synthesis of DNA, RNA and proteins compared to untreated controls. An inhibition of 30‒50% was observed in *S. aureus* (Fig. 5B,C and D); increased label incorporation into peptidoglycans was also observed (Fig. 5A). Conversely, treatment with Lfcin5 appeared to promote all macromolecular biosynthetic pathways examined in *S. aureus* ATCC25923 cells (Fig. 5) but requires further study.

### Efficacy of Lfcin4 and Lfcin5 *in vivo*

#### Murine thigh infection model

**Figure 6:**
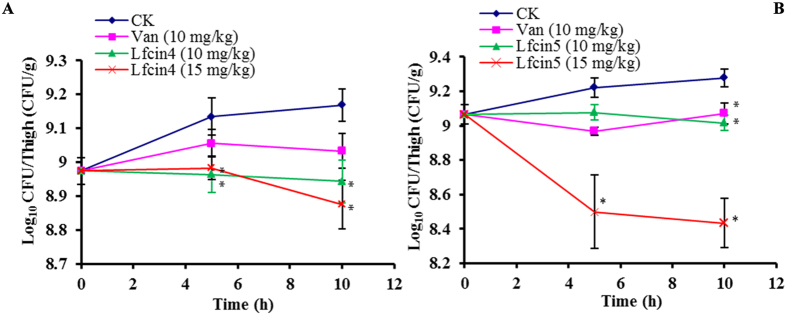
Efficacy of Lfcin4 and Lfcin5 against *S. aureus* ATCC25923 in the neutropenic murine thigh infection model. **(A)** Lfcin4 treatment. **(B)** Lfcin5 treatment. Control: mice treated with saline only; Van (10 mg/kg): mice treated with a single intravenous (tail) dose of vancomycin (10 mg/kg); Lfcin4/Lfcin5 (10 mgkg) and Lfcin4/Lfcin5 (15 mg/kg): mice treated with a single intravenous (tail) dose of 10 or 15 mg/kg Lfcin4 or Lfcin5, respectively. Results are presented as the mean ± SEM. Differences between groups were determined by one-way ANOVA followed by SPSS analysis (n = 4 per group). *p < 0.05 compared to the control group.

The neutropenic mouse thigh infection model has been extensively used to evaluate the *in vivo* antimicrobial activity of AMPs. The viable bacterial counts from infected thighs after treatment with peptides are shown in Fig. 6. Both Lfcin4 and Lfcin5 were effective at reducing the bacterial load in treated mice. At the start of therapy, in treated mice, 8.97 (the Lfcin4 group) and 9.07 (the Lfcin5 group) log_10_ CFU/g of *S. aureus* ATCC25923 were detected in the thigh. Five hours after treatment, the bacterial counts in mice treated with Lfcin4 and Lfcin5 were all reduced by −0.01‒0.10 and −0.01‒0.57 log_10_ CFU/g, respectively, compared to untreated mice (Fig. 6). Ten hours after treatment, an increase of 0.19‒0.21 log_10_ CFU/g was detected in untreated control mice. Treatment with 10 or 15 mg/kg Lfcin4 resulted in a reduced bacterial burden by 0.03 and 0.10 log_10_ CFU/g, respectively. This reduction was less than that observed following vancomycin treatment (−0.06 log_10_ CFU/g) (Fig. 6A). At 5 h, treatment with 15 mg/kg Lfcin5 reduced the bacterial count by 0.57 log_10_ CFU/g. Treatment with 10 mg/kg vancomycin resulted in a decrease of 0.1 log_10_ CFU/g (Fig. 6B). At 10 h post-treatment with 15 mg/kg Lfcin5, approximately 76.6% of *S. aureus* cells had been killed. This indicates that treatment with 15 mg/kg Lfcin5 is more effective at reducing the bacterial burden than treatment with 10 mg/kg vancomycin (−1.2%) and Lfcin4 (20.5%) (Fig. 6B).

#### Lfcin4 and Lfcin5 protect mice from lethal challenge with LPS

**Figure 7:**
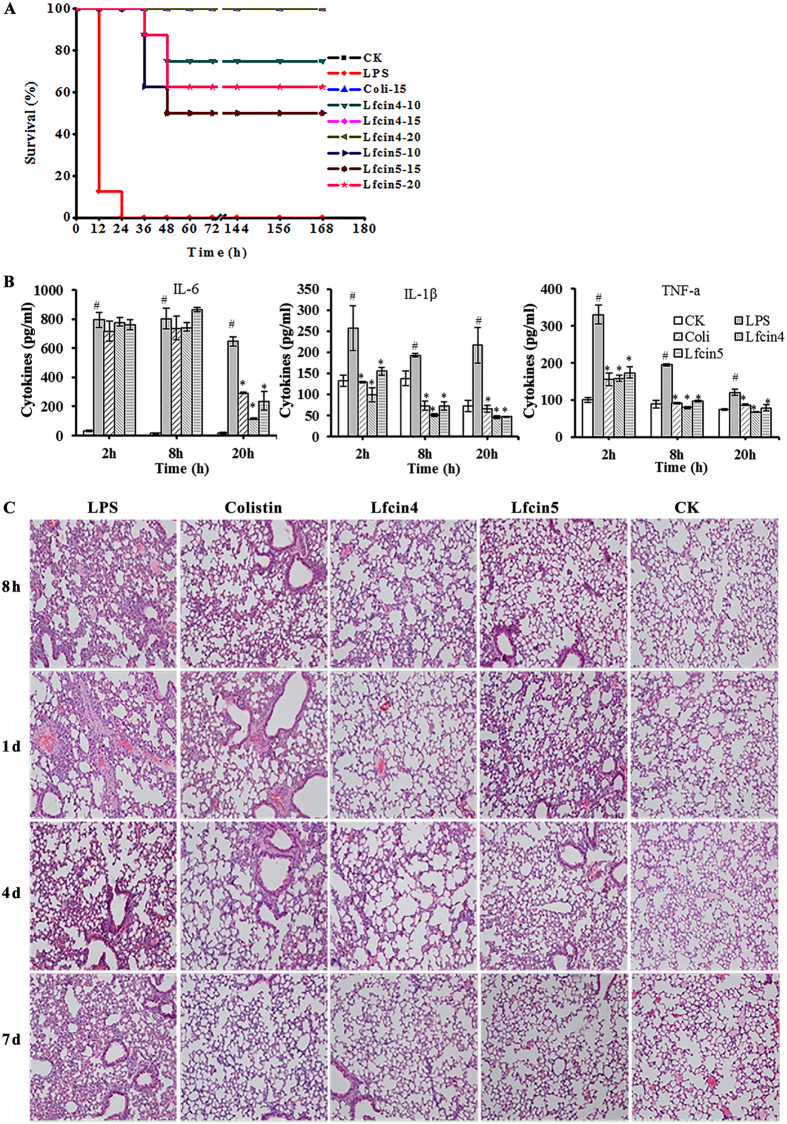
Effects of Lfcin4 and Lfcin5 on LPS-induced responses *in vivo*. **(A)** Groups of eight C57BL/6 mice were intraperitoneally injected with LPS from *E. coli* O111:B4 (30 mg/kg of body weight). Thirty minutes after LPS injection, Lfcin4, Lfcin5, colistin, or saline was administered intraperitoneally. Survival was recorded every 12 h and followed for up to 7 d. **(B,C)** C57BL/6 mice were intraperitoneally injected with 10 mg/kg LPS followed by intraperitoneal administration of 15 mg/kg Lfcin4, Lfcin5 or colistin 30 min later. Mice treated with buffer only served as a control. Cytokines were measured in blood from mice sacrificed at 2 h, 8 h or 20 h after LPS injection. The results are presented as the mean ± SEM. Differences between groups were determined by one-way ANOVA followed by SPSS analysis (n = 3 per group). ^#^p < 0.05 compared to the control group and *p < 0.05 compared to the LPS-treated group **(B)**. Light microscopy images (10 × magnification, scale bar: 100 μm) of lung tissue from representative mice sacrificed at 8 h, 1 d, 4 d, and 7 d after LPS injection **(C)**.

To evaluate the therapeutic activity of peptides in the endotoxemia model, mice were injected with colistin (15 mg/kg) or Lfcin4 or Lfcin5 (10, 15 and 20 mg/kg) at 0.5 h, 8 h and 24 h after challenge with LPS. Mice in the control group were injected with PBS instead of LPS and survived the experimental period (Fig. 7A). The mice injected with LPS began to die 12 h after inoculation; all mice had succumbed within 24 h. Treatment with 10, 15 or 20 mg/kg Lfcin4 improved the survival rates to 75%, 100% and 100%, respectively. Mice treated with 10, 15 or 20 mg/kg Lfcin5 responded in a dose-dependent fashion, with survival rates of 50%, 62.5% and 75%, respectively. The survival rate of mice treated with 15 mg/kg colistin was 75% (Fig. 7A). These results indicate that both Lfcin4 and Lfcin5 protect mice from lethal LPS challenge *in vivo*.

To determine whether the protective activities of Lfcin4 and Lfcin5 are associated with inflammatory cytokines, the serum levels of proinflammatory cytokines interleukin-6 (IL-6), interleukin-1β (IL-1β) and tumor necrosis factor-α (TNF-α) were determined in endotoxemic mice at different time points using an enzyme-linked immunosorbent assay (ELISA). As shown in Fig. 7B, the concentrations of serum IL-6, IL-1β and TNF-α were 779.5‒115.8, 100.1‒6.3 and 158.9‒67.8 pg/ml, respectively, for endotoxemic mice treated with Lfcin4 and were 761.6‒240.2, 155.2‒47.7 and 173.8‒78.3 pg/ml, respectively, for mice treated with Lfcin5. The levels of IL-1β and TNF-α were significantly lower than those from the corresponding LPS control groups (717.2‒294.9, 258.0‒193.2 and 330.3‒120.5 pg/ml, respectively) and the colistin groups. These data suggest that treatment with either Lfcin4 or Lfcin5 inhibits the production of proinflammatory cytokines.

To determine whether Lfcin4 or Lfcin5 protects mice from LPS-induced lung injury, the lungs of mice were examined histologically at 8 h‒7 d after treatment. As shown in Fig. 7C, no pathological change was found in the lungs of mice not injected with LPS, whereas LPS-challenged mice treated with saline developed acute lung injury, characterized by alveolar septum distention, inflammatory cells, an infiltration of red blood cells, and a certain degree of lung tissue distortion. In contrast, after treatment with Lfcin4 or Lfcin5, there was apparently less lung damage at 8 h‒4 d and no obvious pathological differences in the lung tissues were observed at 7 d. The efficacy of both Lfcin4 and Lfcin5 was higher than that of colistin. These data demonstrate that, similar to colistin, both Lfcin4 and Lfcin5 protect mice from lethal LPS challenge *in vivo*.

## Discussion

Lfcin is a multifunctional peptide that targets antibiotic-resistant bacterial strains, fungi, viruses, parasites, and tumors, as well as directly binds to the bacterial endotoxin LPS and is thereby already being used in different preclinical and clinical trials^11^. In this work, we designed five 17-residue LfcinB17-31 derivatives (Trp-rich and Ala-rich) and first studied their antibacterial activities; Lfcin4 and Lfcin5 were found to be the most active peptides among those tested against both *S. aureus* and *S. enteritidis*, displaying more than 2- to 8-fold increases in antibacterial activity over the parent peptide, Lfcin1 (LfcinB17-31) (Table 2).

The α-helical conformation is necessary for the activity of some AMPs such as magainin and cecropin^35^,^36^. In this study, there was also a positive correlation between the α-helical content and antibacterial activity of LfcinB17-31 derivatives. Both Lfcin4 and Lfcin5 had the highest α-helix content at 64.7% and the most potent antimicrobial activities (Tables 1 and 2), which may be related to Ala at positions 3, 7 and 14 since Ala is favorable for α-helix formation^37^. Conversely, Lfcin6, with the lowest α-helical content, was inactive, wherein Arg and Met modifications in positions 4 and 10 by Trp resulted in a decrease in α-helix content from 60% to 35.2% (Tables 1 and 2). Furthermore, the CD spectra verified that both Lfcin4 and Lfcin5 contain an amphipathic α-helical structure (Fig. 1B and C, Supplementary Table S2). Therefore, it appears that the α-helical structure of the LfcinB17-31 derivatives is the most important structural parameter affecting antibacterial activity, while the antimicrobial activity of these peptides was less dependent on their positive charge.

Rekdal *et al*. reported that an increase in hydrophobicity leads to an increase in antibacterial activity^18^. In our study, the enhanced activity of certain peptides may be due to the increased hydrophobicity by the replacement of Cys, Arg, Gln, Met, and Gly residues at positions 3, 4, 7, 10, and 14 in Lfcin1 by an aliphatic Ala or an aromatic Trp or Phe with larger hydrophobic side chains, which contribute largely to the depth of penetration into the hydrophobic core of a bilayer and the strength of interaction with negatively charged membranes, respectively^38^. Lfcin4 and Lfcin5 contain the largest number of hydrophobic residues. These results are consistent with previous conclusions that the increase in activity of Lfcin peptides (LFB A3, LFB Q7 and LFB G14) may be due to increased hydrophobicity that comes with the replacement of Cys, Gln or Gly residues by Ala^20^. Additionally, Trp is essential for the pore-forming properties of the peptide^27^. LfcinB uses Trp solely as a hydrophobic anchoring force to help thread the peptide through the membrane by its hydrophobic character^21^. However, it is also evident that there is an upper limit to the number of Trp residues that is tolerated, as peptide Lfcin6 (containing four Trp residues at positions 3, 6, 8, and 10) in this study exhibited much less antimicrobial activity than Lfcin4 (containing three Trp residues at positions 4, 6 and 8) and Lfcin5 (containing three Trp residues at positions 6, 8 and 10). This finding is in agreement with a previous study that reported that the LFB derivatives containing three additional Trp residues had lower antimicrobial activity than the LFB peptide with two Trp residues^21^. Cation-π interactions and hydrogen bonds occur mainly between aromatic residues (e.g., Trp and Phe) and residues with positively charged side chains (e.g., Arg and Lys), which may allow the peptides to penetrate deeper into the membrane^22^. All of these factors combined (i.e., α-helical content, hydrophobicity and intermolecular interactions) may have contributed to a more efficient activity of Lfcin4 and Lfcin5 against bacteria than their parent peptide.

The LfcinB peptide exerts a minor permeabilizing effect on the cytoplasmic membrane of susceptible bacteria; however, it does not lyse the bacteria^25^,^39^. In our study, both Lfcin4 and Lfcin5 induced a concentration-dependent leakage of entrapped calcein, with the former having more potent interactions with negatively charged phospholipids than the latter. Additionally, the effects of Lfcin4 and Lfcin5 on Gram-negative bacteria differ from those on Gram-positive bacteria. Compared to Lfcin5, Lfcin4 demonstrates relatively stronger outer membrane permeabilization capacity against *S. aureus* and *S. enteritidis* (Fig. 3B and C). Moreover, Lfcin4 permeabilized the inner membrane of *S. aureus* and induced the leakage of cytoplasmic content, although Lfcin5 did not cause any significant damage to the integrity of the inner membrane; only shrinkage of cells was observed (Figs 2C and 3E). It is very interesting that the effect of Lfcin5 on *S. enteritidis* was more rapid and more profound than that of Lfcin4 (Fig. 3F and G), as observed by SEM (Fig. 2C). These results suggest a possible intracellular target for Lfcin4 and Lfcin5.

The interactions between the peptides and bacterial genomic DNA examined in our study imply that both Lfcin4 and Lfcin5 bind to genomic DNA from *S. aureus, S. enteritidis* and *E. coli* (Fig. 4). This result is contrary to the previous finding that LfcinB did not bind to pGEM-βGAL plasmid DNA^25^. Moreover, we found that Lfcin4 inhibited DNA, RNA and protein synthesis in *S. aureus* whereas Lfcin5 did not (Fig. 5), which is similar to LfcinB17-41 and other Trp-rich peptides derived from human lysozymes that have been shown to translocate across lipid bilayers and inhibit macromolecular synthesis^25^,^39^. Therefore, it appears that Lfcin4 and Lfcin5 use multiple mechanisms to kill bacteria.

Conversely, in this study, Lfcin4 and Lfcin5 demonstrated excellent *in vivo* activity against *S. aureus* in mice (Fig. 6), as does human Lfcin1-11^40^. A dose-dependent effect was observed, where increasing doses of Lfcin4 and Lfcin5 from 10 to 15 mg/kg of body weight resulted in a greater reduction of viable bacteria in infected thighs of 0.85 log_10_ CFU/g of tissue compared to control mice (Fig. 6). We also showed that both Lfcin4 and Lfcin5 increased the survival of endotoxemic mice and protected the lungs from acute injury induced by LPS and that they were more effective than colistin (Fig. 7A and C). Moreover, these peptides significantly inhibited the release of cytokines (IL-6, IL-1β and TNF-α) in mice challenged with LPS (Fig. 7B). Together, these results suggest that both Lfcin4 and Lfcin5 are potential endotoxemia therapeutics.

In conclusion, we designed a series of LfcinB17-31 derivatives based on net charge, α-helical content and hydrophobicity. Both Lfcin4 and Lfcin5 showed stronger antimicrobial activity than the parent peptide and had very low hemolysis. Lfcin4 and Lfcin5 disrupt bacterial cell membranes and interact with bacterial DNA. DNA, RNA and protein synthesis were inhibited in *S. aureus* by Lfcin4 but not by Lfcin5. Both Lfcin4 and Lfcin5 protected mice from *S. aureus* infection and from LPS-induced lethality. Our findings strongly suggest that Lfcin4 and Lfcin5 (Trp-rich and Ala-rich) could serve as promising antimicrobial and anti-endotoxin agents for further clinical applications.

## Materials and Methods

### Peptide design

A series of LfcinB17-33 derivatives was designed based on several structural parameters, including net charge, α-helical content and hydrophobicity, by replacing the Cys residue at position 3 with Ala, Arg4 with Phe or Trp, Glu7 with Ala, Met10 with Trp, and Gly14 with Ala (Table 1). The physicochemical properties of these peptides were determined using the ProtParam tool on the ExPASy server (http://web.expasy.org/protparam/) and the Antimicrobial Peptide Calculator and Predictor tool from the APD database (http://aps.unmc.edu/AP/prediction/prediction_main.php). The α-helical index was calculated by NPS@ (http://npsa-pbil.ibcp.fr/cgi-bin/npsa_automat.pl?page=npsa_hnn.html). The structures of the peptides were predicated by Emboss explorer (http://emboss.bioinformatics.nl/) and the I-TASSER sever (http://zhanglab.ccmb.med.umich.edu/I-tasser). Electrostatic surface potential was evaluated by PyMOL. All peptides were over 95% pure and were synthesized by SBS Genetech Co., Ltd. (Beijing, China) and ChinaPeptides Co., Ltd. (Shanghai, China), respectively.

### Antibacterial and hemolytic activity

The MIC values of LfcinB17-33 derivatives against bacteria were determined by the microtiter broth dilution method^41^. Briefly, 10 μl of serially diluted aliquots of peptides were added to each well of 96-well microtiter plates, followed by the addition of 90 μl exponential phase bacteria (10^5^ CFU/ml) and incubated for 16‒18 h at 37 °C. The MIC value was determined as the lowest peptide concentration at which the peptide completely inhibited the growth of bacteria.

The hemolytic activities of Lfcin4 and Lfcin5 were evaluated based on the amount of released hemoglobin from erythrocyte suspensions of healthy mouse blood according to a previous method^42^. Blood cells were centrifuged at 2000 rpm for 5 min at 4 °C and washed three times with a saline solution (0.9% NaCl). Erythrocytes were resuspended in 0.9% NaCl, and 100 μl aliquots were mixed with 100 μl of peptides at different concentrations ranging from 2 to 1024 μg/ml. The mixtures were then added to 96-well plates. After incubation at 37 °C for 1 h, cells were centrifuged at 5000 rpm for 5 min, and the absorbance of the supernatant was measured at 540 nm on a microtiter plate reader. Controls for 0% and 100% hemolysis release were determined with 0.9% NaCl (A_NC_) and 0.1% Triton X-100 (A_PC_), respectively. Hemolytic percentages are expressed as follows: hemolysis (%) = [(A_sample_ − A_NC_/(A_PC_ − A_NC_)] ×100%, where A_sample_ is the absorbance value of each sample. All experiments were performed at least three times.

### CD analysis

The secondary structures of Lfcin4 and Lfcin5 were investigated in ddH_2_O, a 50% TFE buffer and an SDS solution by CD spectroscopy. The CD spectra of Lfcin4 and Lfcin5 were determined on an MOS-450 spectropolarimeter (Bio-Logic, Grenoble, France) as previously described^42^. Peptides were dissolved in ddH_2_O, 5‒40 mM SDS or a 50% TFE buffer. Samples were loaded into a quartz cuvette (1.0 mm path length), and spectra were recorded from 180 to 260 nm at 25 °C with a step resolution of 2.0 nm, a scanning speed of 100 nm/min and an integration time of 2 s.

### Cell surface hydrophobicity assay

The effects of AMPs on the cell surface hydrophobicity of *S. aureus* ATCC25923 and *S. enteritidis* CVCC3377 were measured using the hexadecane partitioning method^41^. Cells were grown to stationary phase in Mueller-Hinton broth at 37 °C, harvested by centrifugation at 5000 rpm for 4 min, washed twice with sterile normal saline, and suspended in 0.1 M KNO_3_ (pH 6.2) to an OD_600 nm_ of 0.4. Lfcin4 or Lfcin5 was added to a final concentration of 1×, 2× or 4 × MIC and incubated for 10 min at 37 °C. Sterile normal saline was used as a negative control, and the optical density at 600 nm was measured as OD_0_. Subsequently, a 1.2-ml cell suspension was mixed with 0.2 ml of hexadecane by vortexing for 1 min, and then, the mixture was incubated for 15 min. The OD_600 nm_ value of the aqueous phase was determined as OD_1_. The hydrophobicity percentages were calculated by the following equation: hydrophobicity (%) = (1 − OD_1_/OD_0_) × 100%. All experiments were repeated three times.

### SEM observations

Mid-log phase *S. aureus* ATCC25923 and *S. enteritidis* CVCC3377 suspensions (1 × 10^8^ CFU/ml) were incubated with 1 × MIC Lfcin4 or Lfcin5 for 2 h at 37 °C. The samples were centrifuged at 4000 rpm for 5 min, washed three times with 0.1 M PBS (pH 7.2) and fixed with 2.5% glutaraldehyde at 4 °C for 2 h. Subsequently, the cells were washed three times with 0.1 M PBS and post-fixed with 1% osmium tetroxide (OsO_4_) for 2 h. The samples were dehydrated through a graded ethanol series (50–70–85–95–100%), CO_2_-dried, followed by platinum coating and then observed under a QUANTA200 SEM (FEI, Philips, Netherlands).

### Membrane permeability assay

#### Effects of Lfcin4 and Lfcin5 on model membranes

Large unilamellar vesicles (LUVs) composed of POPC/POPG (1:3) were used as a model anionic membrane^43^. LUVs were dissolved in chloroform, dried under nitrogen gas and vacuum-dried for 2 h. The lipid film was rehydrated in 20 mM HEPES (150 mM NaCl, 1 mM EDTA, pH 7.4). To make calcein-encapsulated unilamellar liposomes, the lipid film was suspended in 5 mM sodium HEPES (containing 100 mM calcein, pH 7.5). The liposome suspensions were freeze-thawed in liquid nitrogen five times and extruded ten times through two stacked polycarbonate filters (100 nm pore size) in an Avanti Mini-Extruder. Untrapped calcein was removed using a Sephadex G-50 column (Amersham Pharmacia Biotech, Little Chalfont, UK). Peptides were then added to the LUVs. After a 10-min incubation at room temperature, the release of calcein form vesicles was monitored for 10 min on a Tecan Infinite M200 PRO plate reader (TECAN, Austria) with an excitation wavelength of 485 nm and an emission wavelength of 535 nm. The total calcein fluorescence (F_T_) was determined by the addition of 0.1% Triton X-100. Dye leakage was calculated according to the following equation: leakage rate (%) = ((F − F_0_)/(F_T_ − F_0_)), where F_0_ indicates the fluorescence of each sample at T = 0.

#### Effects of Lfcin4 and Lfcin5 on the outer membrane

The effects of Lfcin4 and Lfcin5 on the outer membrane of *S. aureus* ATCC25923 and *S. enteritidis* CVCC3377 were determined by measuring the uptake of NPN^44^,^45^. Mid-log phase cells were washed with sterile normal saline three times and suspended in 5 mM HEPES buffer (pH 7.2, containing 5 mM glucose) to an OD at 600 nm of 0.5. Subsequently, NPN (10 mM) and peptide (1×, 2× or 4 × MIC) solutions were added to 96-well plates. The relative fluorescence intensity was measured using an Infinite M200 PRO plate reader. The excitation and emission wavelengths were set at 328 and 438 nm, respectively. Treatment with PBS severed as a negative control, and treatment with ampicillin or colistin was used as a positive control.

#### Effects of Lfcin4 and Lfcin5 on the inner membrane

The permeabilization of the inner membrane was performed according to a previous report by Li *et al*. with slight modifications^33^. *S. aureus* ATCC25923 and *S. enteritidis* CVCC3377 cells in mid-log phase were washed with 10 mM PBS buffer three times, resuspended in the same buffer (1 × 10^8^ CFU/ml), and incubated with or without 1 × MIC peptides at 37 °C for 5, 30 and 120 min. Bacterial cells were then washed twice with PBS, fixed with 50 μg/ml PI and analyzed using a BD FACS Calibur Flow Cytometer (Franklin Lakes, New Jersey, USA). Data were analyzed using CellQuest Pro software (BD, USA).

### Effects of Lfcin4 and Lfcin5 on bacterial genomic DNA

Genomic DNA was extracted from *S. aureus* ATCC25923, *E. coli* O157 and *S. enteritidis* CVCC3377 using a TIANamp Bacteria DNA Kit (TIANGEN Biotech Co., Ltd., Beijing). The gel retardation experiment was performed by mixing the bacterial DNA with different concentrations of Lfcin4 or Lfcin5 in 20 μl binding buffer (10 mM Tris-HCl, pH 8.0, 5% glycerol, 1 mM dithiothreitol, 1 mM EDTA, 20 mM KCl, and 50 μg/ml bovine serum albumin)^41^. The ratios of peptide to DNA were 0, 0.5, 1, 2.5, 5.0, and 10.0 (w/w). The mixtures were incubated for 10 min at 37 °C and then subjected to electrophoresis on a 0.7% agarose gel.

To examine whether peptide binding causes secondary structure changes in bacterial DNA, CD measurements were carried out on an MOS-450 spectropolarimeter using a quartz cuvette with a 1.0 mm path length. The ratios of peptide to bacterial DNA were 0, 5 and 10. The spectra were recorded from 220 to 320 nm at 25 °C. The CD data represent the average of 10 scans with a 20 s bandwidth.

### Effects of Lfcin4 and Lfcin5 on macromolecular synthesis

The effects of each peptide on the incorporation of L-[methyl-^3^H] thymidine, [5-^3^H] uridine, D-[6-^3^H(N)] glucosamine hydrochloride, and L-[3,4,5-^3^H] leucine into DNA, RNA, peptidoglycans, and proteins were investigated in *S. aureus* ATCC25923. Mid-log phase *S. aureus* cells (10^5^ CFU/ml) were incubated with 1 × MIC peptide or antibiotics at 37 °C for 15 min. Radioactive precursors of ^3^H-thymidine, ^3^H-uridine, ^3^H-leucine, and ^3^H-glucosamine (40 μCi/ml) were added to cultures to measure macromolecular synthesis and incubated for 20 min at 37 °C. Cold 25% trichloroacetic acid (TCA) was added to the mixture and placed on ice for 30 min. After centrifugation, pellets were washed twice with 25% TCA, dried and counted with scintillation fluid on a MicroBeta 1450 scintillation counter (Perkin Elmer)^46^.

### *In vivo* experiments

Animal care and all experimental protocols were approved by the Laboratory Animal Ethical Committee and its Inspection of the Feed Research Institute of Chinese Academy of Agricultural Sciences (AEC-CAAS-20090609) and were performed in accordance with ARRIVE guidelines^47^.

#### Thigh infection model

The thigh infection protocol was performed as previously described^48^. Female BALB/c mice (6 weeks old, 22 ± 2 g) were rendered neutropenic (neutrophils, 100/mm^3^) by intraperitoneal injection with 150 and 100 mg cyclophosphamide per kg of body weight for 4 d and 1 d before infection, respectively. Mice were inoculated with 100 μl of *S. aureus* ATCC25923 cells (1.0 × 10^7^ CFU/ml) and given a single dose (0.2 ml) of Lfcin4 or Lfcin5 (10 or 15 mg/kg of body weight) or vancomycin (10 mg/kg of body weight) by tail vein injection at 2 h post-infection. Mice were humanely euthanized by cervical dislocation at 5 and 10 h after treatment. Thighs were removed and homogenized in sterile PBS for a CFU assay for *S. aureus* ATCC25923.

#### LPS-induced endotoxemia model

Male C57BL/6 mice (8 weeks old, 22 ± 2 g) were intraperitoneally injected with 30 mg/kg of LPS (0.2 ml) from *E. coli* O111:B4 cells as previously described^6^,^49^. Mice were intraperitoneally injected with Lfcin4 or Lfcin5 (10, 15 or 20 mg/kg of body weight in 0.2 ml) or colistin (15 mg/kg of body weight in 0.2 ml) at 0.5, 8 and 24 h after inoculation with LPS, respectively. Mice injected with LPS or saline only served as positive or mock-treated controls, respectively. Survival was recorded every 12 h and followed for up to 7 d.

Serum was separated from mice at 2, 8 and 20 h following the administration of LPS (10 mg/kg). The levels of IL-6, IL-1β and TNF-α in endotoxemic mice were detected at Jiaxuan Biotech. Co., Ltd. (Beijing, China) using an ELISA kit according to the manufacturer’s protocol.

Thirty minutes after intraperitoneal injection of LPS (10 mg/kg of body weight in 0.2 ml), mice were intraperitoneally injected with Lfcin4, Lfcin5 or colistin (15 mg/kg of body weight in 0.2 ml). Mice were sacrificed at 8 h, 1 d, 4 d, and 7 d after LPS injection. The lungs were removed, washed with PBS and fixed in 4% paraformaldehyde at 4 °C for 24 h. After they were washed with PBS and dehydrated with a graded series of ethanol (75‒95%), the tissues were infiltrated with xylene and embedded in paraffin wax. Sections were cut, stained with hematoxylin and eosin and examined under a light microscope. Mice injected with LPS or only PBS served as positive or mock-treated controls, respectively.

### Statistical analysis

Statistical analysis was performed using software SPSS21.0 (SPSS, USA). Data are expressed as the mean ± standard error of the mean (SEM). One-way repeated analysis of variance (ANOVA) (Institute Inc., Cary, NC, USA), Duncan method and the Mann-Whitney rank test were used to determine the statistical significance. A p value of <0.05 was considered statistically significant.

## Additional Information

**How to cite this article**: Hao, Y. *et al*. Killing of *Staphylococcus aureus* and *Salmonella enteritidis* and neutralization of lipopolysaccharide by 17-residue bovine lactoferricins: improved activity of Trp/Ala-containing molecules. *Sci. Rep.* **7**, 44278; doi: 10.1038/srep44278 (2017).

**Publisher's note:** Springer Nature remains neutral with regard to jurisdictional claims in published maps and institutional affiliations.

## Supplementary Material

Supplementary Information

## Figures and Tables

**Table 1:**
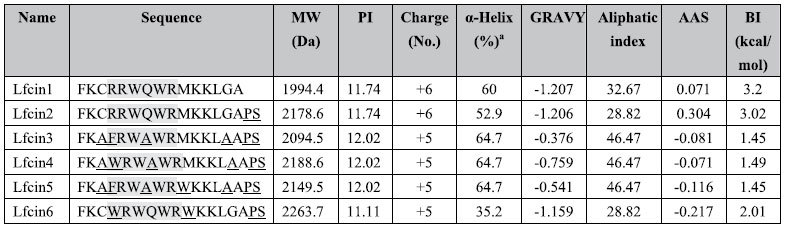
Amino acid sequences and physicochemical properties of LfcinB17-31 and its derivatives.

Abbreviations: Lfcin1: LfcinB17-31; MW: molecular weight; PI: isoelectric point; GRAVY: grand average of hydropathicity; shadowed residues: the active core; underlined residues: substitutions or additional residues; AAS: antibacterial activity score; BI: Boman index. a: calculated by NPS@.

**Table 2:** MIC values of LfcinB17-31 and its derivatives against bacteria.

Strains	MIC											
	Lfcin1		Lfcin2		Lfcin3		Lfcin4		Lfcin5		Lfcin6	
	μM	μg/ml	μM	μg/ml	μM	μg/ml	μM	μg/ml	μM	μg/ml	μM	μg/ml
Gram-negative bacteria												
* Escherichia coli* CVCC195^a^	16	32	15	32	31	64	30	64	30	64	57	128
* E. coli* CVCC1515^a^	16	32	>59	>128	31	64	15	32	60	128	57	128
* E. coli* CICC21530 (serotype O157:H7)^b^	32	64	59	128	31	64	15	32	30	64	57	128
* Salmonella typhimurium* ATCC14028^c^	16	32	29	64	61	128	58	128	>30	>64	>57	>128
* S. enteritidis* CVCC3377^a^	8	16	15	32	1.9	4	1.8	4	1.9	4	7.1	16
* S. pullorum* CVCC1789^a^	>64	>128	>59	>128	>61	>128	>58	>128	>60	>128	>57	>128
* S. pullorum* CVCC1802^a^	>64	>128	>59	>128	>61	>128	>58	>128	>60	>128	>57	>128
* S. choleraesuis* CVCC503^a^	64	128	59	128	61	128	58	128	60	128	>57	>128
* Pseudomonas aeruginosa* CICC10419^b^	>64	>128	>59	>128	>61	>128	>58	>128	>60	>128	>57	>128
* P. aeruginosa* CICC21630^b^	>64	>128	>59	>128	>61	>128	>58	>128	>60	>128	>57	>128
Gram-positive bacteria												
* Staphylococcus aureus* ATCC43300^c^	>64	>128	>59	>128	>61	>128	29	64	30	64	>57	>128
* S. aureus* ATCC25923^c^	1	2	0.92	2	0.95	2	3.66	8	0.93	2	7.1	16
* Streptococcus suis* CVCC606^a^	>64	>128	>59	>128	61	128	58	128	60	128	>57	>128
* Enterococcus faecium* CMCC1.2136^d^	8	16	15	32	15	32	7.3	16	7.4	16	14	32
* Bacillus subtilis* ATCC6633^c^	32	64	29	64	15	32	15	32	15	32	57	128

^a^China Veterinary Culture Collection Center (CVCC).

^b^China Center of Industrial Culture Collection (CICC).

^c^American Type Culture Collection (ATCC).

^d^National Center for Medical Culture Collection (CMCC). The data are representative of three independent experiments.

## References

[b1] Diekema D. J. . Survey of infections due to *Staphylococcus* species: frequency of occurrence and antimicrobial susceptibility of isolates collected in the United States, Canada, Latin America, Europe, and the Western Pacific region for the SENTRY Antimicrobial Surveillance Program, 1997-1999. Clin. Infect. Dis. 32 Suppl 2, S114–S132 (2001). 11320452 10.1086/320184

[b2] Wang Z., Cao B., Liu Y. M., Gu L. & Wang C. Investigation of the prevalence of patients co-colonized or infected with methicillin-resistant *Staphylococcus aureus* and vancomycin- resistant enterococci in China: a hospital-based study. Chin. Med. J. (Engl.) 122, 1283–1288 (2009). 19567138

[b3] Maiti S. . Effective control of *Salmonella* infections by employing combinations of recombinant antimicrobial human β-defensins hBD-1 and hBD-2. Antimicrob. Agents Chemother. 58, 6896–6903 (2014). 25199778 10.1128/AAC.03628-14 PMC4249419

[b4] Lu Q. . Pathogen and antimicrobial resistance profiles of culture-proven neonatal sepsis in Southwest China, 1990-2014. J. Paediatr. Child Health 52, 939–943 (2016). 27500793 10.1111/jpc.13278

[b5] Miller M. F. & Loch-Caruso R. Comparison of LPS-stimulated release of cytokines in punch versus transwell tissue culture systems of human gestational membranes. Reprod Biol. Endocrinol. 8, 121 (2010). 20950439 10.1186/1477-7827-8-121 PMC2965156

[b6] Kalle M. . Host defense peptides of thrombin modulate inflammation and coagulation in endotoxin-mediated shock and *Pseudomonas aeruginosa* sepsis. PLoS One 7, e51313 (2012). 23272096 10.1371/journal.pone.0051313 PMC3521733

[b7] Russell S., Hayes M. A., Simko E. & Lumsden J. S. Plasma proteomic analysis of the acute phase response of rainbow trout (*Oncorhynchus mykiss*) to intraperitoneal inflammation and LPS injection. Dev. Comp. Immunol. 30, 393–406 (2006). 16139357 10.1016/j.dci.2005.06.002

[b8] Tomita M. . Potent antibacterial peptides generated by pepsin digestion of bovine lactoferrin. J. Dairy Sci. 74, 4137–4142 (1991). 1787185 10.3168/jds.S0022-0302(91)78608-6

[b9] Bellamy W., Takase M., Wakabayashi H., Kawase K. & Tomita M. Antibacterial spectrum of lactoferricin B, a potent bactericidal peptide derived from the N-terminal region of bovine lactoferrin. J. Appl. Bacteriol. 73, 472–479 (1992). 1490908 10.1111/j.1365-2672.1992.tb05007.x

[b10] Yamauchi K., Tomita M., Giehl T. J. & Ellison R. T. 3rd. Antibacterial activity of lactoferrin and a pepsin-derived lactoferrin peptide fragment. Infect. Immun. 61, 719–728 (1993). 8423097 10.1128/iai.61.2.719-728.1993 PMC302785

[b11] Gifford J. L., Hunter H. N. & Vogel H. J. Lactoferricin: a lactoferrin-derived peptide with antimicrobial, antiviral, antitumor and immunological properties. Cell Mol. Life Sci. 62, 2588–2598 (2005). 16261252 10.1007/s00018-005-5373-z PMC11139180

[b12] Jenssen H. & Hancock R. E. W. Antimicrobial properties of lactoferrin. Biochimie 91, 19–29 (2009). 18573312 10.1016/j.biochi.2008.05.015

[b13] Oo T. Z., Cole N., Garthwaite L., Willcox M. D. & Zhu H. Evaluation of synergistic activity of bovine lactoferricin with antibiotics in corneal infection. J. Antimicrob. Chemother. 65, 1243–1251 (2010). 20375033 10.1093/jac/dkq106

[b14] Liu Y., Han F., Xie Y. & Wang Y. Comparative antimicrobial activity and mechanism of action of bovine lactoferricin-derived synthetic peptides. Biometals 24, 1069–1078 (2011). 21607695 10.1007/s10534-011-9465-y

[b15] Silva T. . Structural diversity and mode of action on lipid membranes of three lactoferrin candidacidal peptides. Biochim. Biophys. Acta 1828, 1329–1339 (2013). 23384417 10.1016/j.bbamem.2013.01.022

[b16] Puknun A. . A heterodimer comprised of two bovine lactoferrin antimicrobial peptides exhibits powerful bactericidal activity against *Burkholderia pseudomallei*. World J. Microbiol. Biotechnol. 29, 1217–1224 (2013). 23404819 10.1007/s11274-013-1284-6

[b17] Wang S. . The effect of Lfcin-B on non-small cell lung cancer H460 cells is mediated by inhibiting VEGF expression and inducing apoptosis. Arch. Pharm. Res. 38, 261–271 (2015). 24691828 10.1007/s12272-014-0373-x

[b18] Rekdal Ø., Andersen J., Vorland L. H. & Svendsen J. S. Construction and synthesis of lactoferricin derivatives with enhanced antibacterial activity. J. Peptide Sci. 5, 32–45 (1999).

[b19] Schibli D. J., Hwang P. M. & Vogel H. J. The structure of the antimicrobial active center of lactoferricin B bound to sodium dodecyl sulfate micelles. FEBS Lett. 446, 213–217 (1999). 10100843 10.1016/s0014-5793(99)00214-8

[b20] Strøm M. B., Rekdal Ø. & Svendsen J. S. Antibacterial activity of 15-residue lactoferricin derivatives. J. Peptide Res. 56, 265–274 (2000). 11095180 10.1034/j.1399-3011.2000.00770.x

[b21] Strøm M. B. . Important structural features of 15-residue lactoferricin derivatives and methods for improvement of antimicrobial activity. Biochem. Cell Biol. 80, 65–74 (2002). 11908644 10.1139/o01-236

[b22] Silva T. . Killing of *Mycobacterium avium* by lactoferricin peptides: improved activity of arginine- and D-amino-acid-containing molecules. Antimicrob. Agents Chemother. 58, 3461–3467 (2014). 24709266 10.1128/AAC.02728-13 PMC4068459

[b23] Vorland L. H., Ulvatne H., Rekdal O. & Svendsen J. S. Initial binding sites of antimicrobial peptides in *Staphylococcus aureus* and *Escherichia coli*. Scand. J. Infect. Dis. 31, 467–473 (1999). 10576125 10.1080/00365549950163987

[b24] Vorland L. H. . Interference of the antimicrobial peptide lactoferricin B with the action of various antibiotics against *Escherichia coli* and *Staphylococcus aureus*. Scand. J. Infect. Dis. 31, 173–177 (1999). 10447328 10.1080/003655499750006236

[b25] Ulvatne H., Samuelsen Ø., Haukland H. H., Krämer M. & Vorland L. H. Lactoferricin B inhibits bacterial macromolecular synthesis in *Escherichia coli* and *Bacillus subtilis*. FEMS Microbiol. Lett. 237, 377–384 (2004). 15321686 10.1016/j.femsle.2004.07.001

[b26] Ho Y. H., Sung T. C. & Chen C. S. Lactoferricin B inhibits the phosphorylation of the two-component system response regulators BasR and CreB. Mol. Cell Proteomics 11, M111.014720 (2012). 10.1074/mcp.M111.014720 PMC3322574 22138548

[b27] Haug B. E. & Svendsen J. S. The role of tryptophan in the antibacterial activity of a 15-residue bovine lactoferricin peptide. J. Pept. Sci. 7, 190–196 (2001). 11354462 10.1002/psc.318

[b28] Kovacs J. M., Mant C. T. & Hodges R. S. Determination of intrinsic hydrophilicity/hydrophobicity of amino acid side chains in peptides in the absence of nearest-neighbor or conformational effects. Biopolymers 84, 283–297 (2006). 16315143 10.1002/bip.20417 PMC2744689

[b29] Cheng D. Q., Li Y. & Huang J. F. Molecular evolution of the primate α-/θ-defensin multigene family. PLoS One 9, e97425 (2014). 24819937 10.1371/journal.pone.0097425 PMC4018336

[b30] Liu Y. F., Xia X., Liang X. & Wang Y. Z. Design of hybrid b-hairpin peptides with enhanced cell specificity and potent anti-inflammatory activity. Biomaterial 34, 237–250 (2013). 10.1016/j.biomaterials.2012.09.032 23046754

[b31] Maillard J. Y. Bacterial target sites for biocide action. Symp. Ser. Soc. Appl. Microbiol. 31, 16S–27S (2002). 12481825

[b32] Xi D. . Mechanism of action of the tri-hybrid antimicrobial peptide LHP7 from lactoferricin, HP and plectasin on *Staphylococcus aureus*. Biometals 27, 957–968 (2014). 25015218 10.1007/s10534-014-9768-x

[b33] Li L., Shi Y., Cheserek M. J., Su G. & Le G. Antibacterial activity and dual mechanisms of peptide analog derived from cell-penetrating peptide against *Salmonella typhimurium* and *Streptococcus pyogenes*. Appl. Microbiol. Biotechnol. 97, 1711–1723 (2013). 22923068 10.1007/s00253-012-4352-1

[b34] Silvestri A. . The interaction of native DNA with Zn(II) and Cu(II) complexes of 5-triethyl ammonium methyl salicylidene orto-phenylendiimine. J. Inorg. Biochem. 101, 841–848 (2007). 17383733 10.1016/j.jinorgbio.2007.01.017

[b35] Bechinger B., Zasloff M. & Opella S. J. Structure and orientation of the antibiotic peptide magainin in membranes by solid-state nuclear magnetic resonance spectroscopy. Protein Sci. 2, 2077–2084 (1993). 8298457 10.1002/pro.5560021208 PMC2142334

[b36] Andreu D., Merrifield R. B., Steiner H. & Boman H. G. N-terminal analogues of cecropin A: synthesis, antibacterial activity, and conformational properties. Biochemistry 24, 1683–1688 (1985). 3924096 10.1021/bi00328a017

[b37] Costantini S., Colonna G. & Facchiano A. M. Amino acid propensities for secondary structures are influenced by the protein structural class. Biochem. Biophys. Res. Commun. 342, 441–451 (2006). 16487481 10.1016/j.bbrc.2006.01.159

[b38] Haney E. F., Nazmi K., Bolscher J. G. & Vogel H. J. Influence of specific amino acid side-chains on the antimicrobial activity and structure of bovine lactoferrampin. Biochem. Cell Biol. 90, 362–377. 22250712 10.1139/o11-057

[b39] Hunter H. N. . The interactions of antimicrobial peptides derived from lysozyme with model membrane systems. Biochim. Biophys. Acta 1668, 175–189 (2005). 15737328 10.1016/j.bbamem.2004.12.004

[b40] Brouwer C. P. & Welling M. M. Various routes of administration of 99mTc-labeled synthetic lactoferrin antimicrobial peptide hLF 1–11 enables monitoring and effective killing of multidrug-resistant *Staphylococcus aureus* infections in mice. Peptides 29, 1109–1117 (2008). 18423795 10.1016/j.peptides.2008.03.003

[b41] Teng D. . A dual mechanism involved in membrane and nucleic acid disruption of AvBD103b, a new avian defensin from the king penguin, against *Salmonella enteritidis* CVCC3377. Appl. Microbiol. Biotechnol. 98, 8313–8325 (2014). 24981062 10.1007/s00253-014-5898-x

[b42] Cho J. & Lee D. G. The characteristic region of arenicin-1 involved with a bacterial membrane targeting mechanism. Biochem. Biophys. Res. Commun. 405, 422–427 (2011). 21241661 10.1016/j.bbrc.2011.01.046

[b43] Pavia K. E., Spinella S. A. & Elmore D. E. Novel histone-derived antimicrobial peptides use different antimicrobial mechanisms. Biochim. Biophys. Acta 1818, 869–876 (2012). 22230351 10.1016/j.bbamem.2011.12.023 PMC3273674

[b44] Han F. F. . Comparing bacterial membrane interactions and antimicrobial activity of porcine lactoferricin-derived peptides. J. Dairy Sci. 96, 3471–3487 (2013). 23567049 10.3168/jds.2012-6104

[b45] Li L., Shi Y., Su G. & Le G. Selectivity for and destruction of *Salmonella typhimurium* via a membrane damage mechanism of a cell-penetrating peptide ppTG20 analogue. Int. J. Antimicrob. Agents 40, 337–343 (2012). 22819152 10.1016/j.ijantimicag.2012.05.026

[b46] Xiong Y. Q., Bayer A. S. & Yeaman M. R. Inhibition of intracellular macromolecular synthesis in *Staphylococcus aureus* by thrombin-induced platelet microbicidal proteins. J. Infect. Dis. 185, 348–356 (2002). 11807717 10.1086/338514

[b47] Kilkenny C., Browne W. J., Cuthill I. C., Emerson M. & Altman D. G. Improving bioscience research reporting: the ARRIVE guidelines for reporting animal research. PLoS Biol. 8, e1000412 (2010). 20613859 10.1371/journal.pbio.1000412 PMC2893951

[b48] Zhang Y. . *In vitro* and *in vivo* characterization of a new recombinant antimicrobial peptide, MP1102, against methicillin-resistant *Staphylococcus aureus*. Appl. Microbiol. Biotechnol. 99, 6255–6266 (2015). 25620367 10.1007/s00253-015-6394-7

[b49] Hu L., Sun C., Wang S., Su F. & Zhang S. Lipopolysaccharide neutralization by a novel peptide derived from phosvitin. Int. J. Biochem. Cell Biol. 45, 2622–2631 (2013). 24028820 10.1016/j.biocel.2013.09.002

