# Supplementary material for: Killing of Staphylococcus aureus and Salmonella enteritidis and neutralization of lipopolysaccharide by 17-residue bovine lactoferricins: improved activity of Trp/Ala-containing molecules
Source: Sci Rep. 2017 Mar 13;7:44278. doi: 10.1038/srep44278 (PMC5347165; doi:10.1038/srep44278)
Supplement: Supplementary Information [file srep44278-s1.pdf]

# Killing of *Staphylococcus aureus* and *Salmonella enteritidis* and neutralization of lipopolysaccharide by 17-residue bovine lactoferricins: improved activity of Trp/Ala-containing molecules

**Ya Hao [1, 2], Na Yang [1, 2], Xiumin Wang [1, 2*], Da Teng [1, 2], Ruoyu Mao [1, 2], Xiao Wang [1, 2], Zhanzhan Li [1, 2] & Jianhua Wang [1, 2*]**

**[1] Key Laboratory of Feed Biotechnology, Ministry of Agriculture, Beijing 100081, China. [2] Gene Engineering Laboratory, Feed Research Institute, Chinese Academy of Agricultural Sciences, Beijing 100081, China.**

*** Correspondence and requests for materials should be addressed to X.M.W. (email: wangxiumin@caas.cn) and J.H.W. (email: wangjianhua@caas.cn; 2681298635@qq.com)**

**SUPPORTING IFORMATION**

**Supplementary 1: Materials and Methods**

**Materials.** *Enterococcus faecalis* CMCC1.2024 was purchased from China Center for Medical Culture Collection (CCMCC). *Escherichia coli* CICC21530 was purchased from China Center of Industrial Culture Collection (CCICC). The other test strains of bacteria shown in Table 2 were purchased from the China Veterinary Culture Collection (CVCC). N-phenyl-1-naphthylamine (NPN), propidiumiodide (PI) and lipopolysaccharides (LPS) from *E. coli* 0111:B4 were purchased from Sigma-Aldrich Chemical Co., Ltd (Shanghai, China). The kits for plasmid and genome DNA extraction were purchased from TIANGEN Biotech (Beijing) Co., Ltd. The antibiotics were purchased from China Institute of Veterinary Drug Control. The other reagents were of analytical grade.

Specific pathogen free (SPF) female BALB/c and male C57BL/6 mice were purchased from the Vital River Laboratories (VRL) (Beijing, China), housed in the appropriate conventional animal care facilities and handled strictly according to international guidelines required for animal experiments.

**Supplementary 2: Tables and Figures**

**Supplementary Table S1** Prediction analysis of the secondary structure of LfcinB17-31 and its derivatives by Emboss explorer.

| Peptides | The percentage of secondary structure (%) | | | |
|---|---|---|---|---|
| | Helix | Turn | Coil | Sheet |
| Lfcin1 | 26.6 | 46.7 | 26.6 | 0 |
| Lfcin2 | 17.6 | 52.9 | 29.4 | 0 |
| Lfcin3 | 64.7 | 5.9 | 29.4 | 0 |
| Lfcin4 | 47.1 | 17.6 | 35.3 | 0 |
| Lfcin5 | 52.9 | 11.8 | 35.3 | 0 |
| Lfcin6 | 0 | 70.6 | 29.4 | 0 |

**Supplementary Table S2** Analysis of the secondary structure of Lfcin4 in different solutions.

| Secondary structure | The percentage of secondary structure of Lfcin4 in different solvents (%) | | | | | |
|---|---|---|---|---|---|---|
| | Lfcin4-$H_2O$ | Lfcin4-5mM SDS | Lfcin4-10mM SDS | Lfcin4-20mM SDS | Lfcin4-40mM SDS | Lfcin4-50% TFE |
| Helix | 11.2 | 17.9 | 12.1 | 12.5 | 12.2 | 17.4 |
| Antiparallel | 35.1 | 24.1 | 34.0 | 33.1 | 34.7 | 27.8 |
| Parallel | 9.2 | 9.3 | 9.2 | 9.2 | 9.0 | 8.9 |
| β-turn | 15.7 | 16.8 | 16.0 | 16.1 | 16.1 | 17.2 |
| Random coli | 28.8 | 31.7 | 28.8 | 29.1 | 28.0 | 28.7 |

**Supplementary Table S3** Analysis of the secondary structure of Lfcin5 in different solutions

| Secondary structure | The percentage of secondary structure of Lfcin5 in different solvents (%) | | | | | |
|---|---|---|---|---|---|---|
| | Lfcin5-$H_2O$ | Lfcin5-5mM SDS | Lfcin5-10mM SDS | Lfcin5-20mM SDS | Lfcin5-40mM SDS | Lfcin5-50% TFE |
| Helix | 11.2 | 21.0 | 11.8 | 12.4 | 12.1 | 17.1 |
| Antiparallel | 34.0 | 19.9 | 34.5 | 34.3 | 33.8 | 28.2 |
| Parallel | 9.0 | 9.4 | 9.1 | 9.0 | 9.2 | 8.8 |
| β-turn | 16.1 | 17.2 | 15.9 | 16.2 | 16.0 | 17.2 |
| Random coli | 28.0 | 32.6 | 28.6 | 28.1 | 28.9 | 28.7 |

**Supplementary Figure S1**

**A**

**Lfcin1**

```
          .   10
      FKCRRWQWRMKKLGA
helix      HHHH
sheet
turns TTTTT    TT
 coil            CCCC
```

**Lfcin2**

```
          .   10
      FKCRRWQWRMKKLGAPS
helix      HHH
sheet
turns TTTTT   TTTT
 coil              CCCCC
```

**Lfcin3**

```
          .   10
      FKAFRWAWRMKKLAAPS
helix HHHHHHHHHH     H
sheet
turns           T
 coil            CCCC C
```

**Lfcin4**

```
          .   10
      FKAWRWAWRWKKLAAPS
helix HHHHHHHH
sheet
turns         TT      T
 coil           CCCCCC
```

**Lfcin5**

```
          .   10
      FKAFRWAWRWKKLAAPS
helix HHHHHHHHH
sheet
turns          T      T
 coil           CCCCCC
```

**Lfcin6**

```
          .   10
      FKCWRWQWRWKKLGAPS
helix
sheet
turns TTTTTTTTTTTT
 coil              CCCCC
```

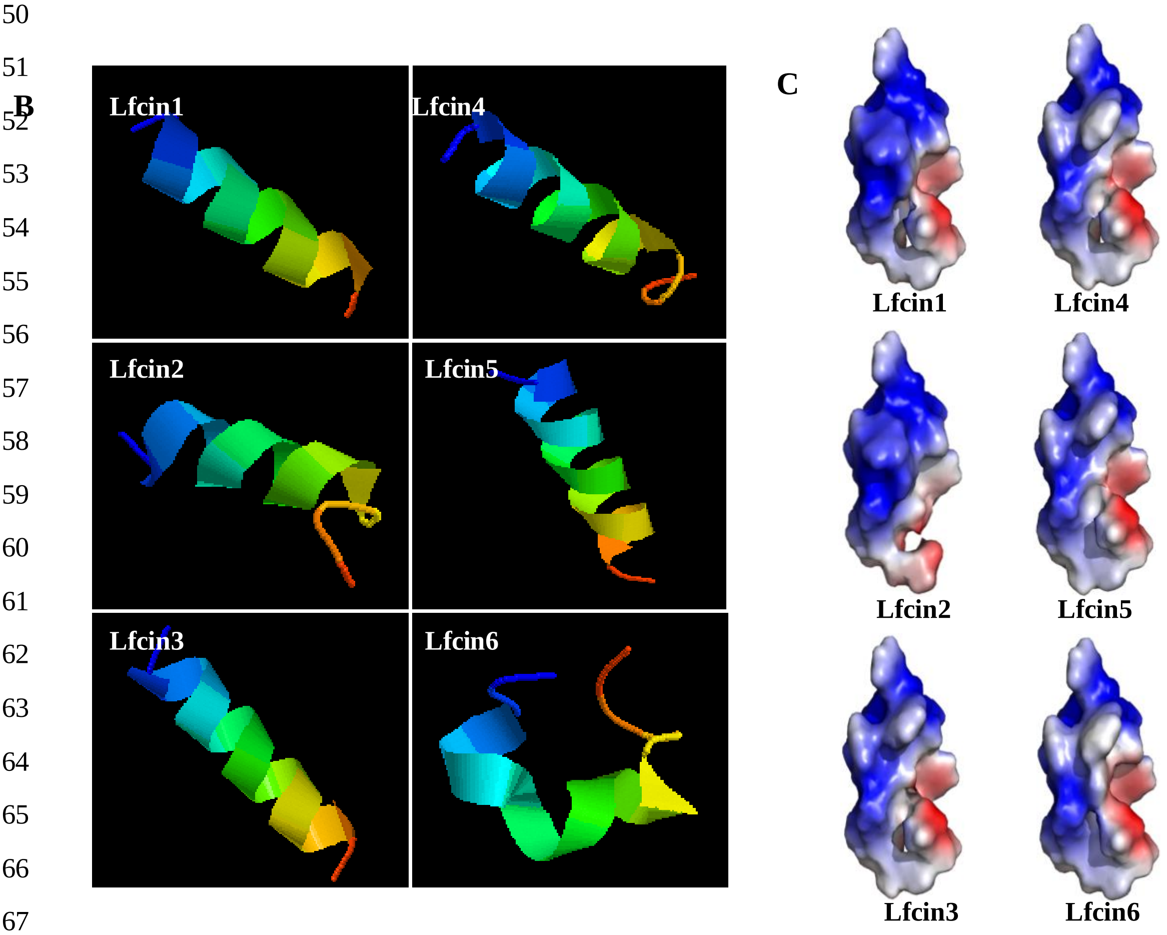


**Supplementary Figure S1 Structures and electrostatic surfaces of LfcinB17-31 and its derivatives.**

**(A)** The 2D molecular modeling generated with Emboss explorer. **(B)** 3D molecular modeling

generated with the I-TASSER sever. **(C)** Electrostatic surface analyzed with PyMOL 1.8. Potential values are given in units of kT per unit charge (k Boltzmann's constant; T temperature). Blue, red and white represent positive, negative, and neutral charge, respectively. The electrostatic potential of Lfcin1, Lfcin2, Lfcin3, Lfcin4, Lfcin5 and Lfcin6 is ±98.263, ±93.267, ±84.244, ±84.611, ±85.553 and ±86.594 kT/e, respectively.

**Supplementary Figure 2**

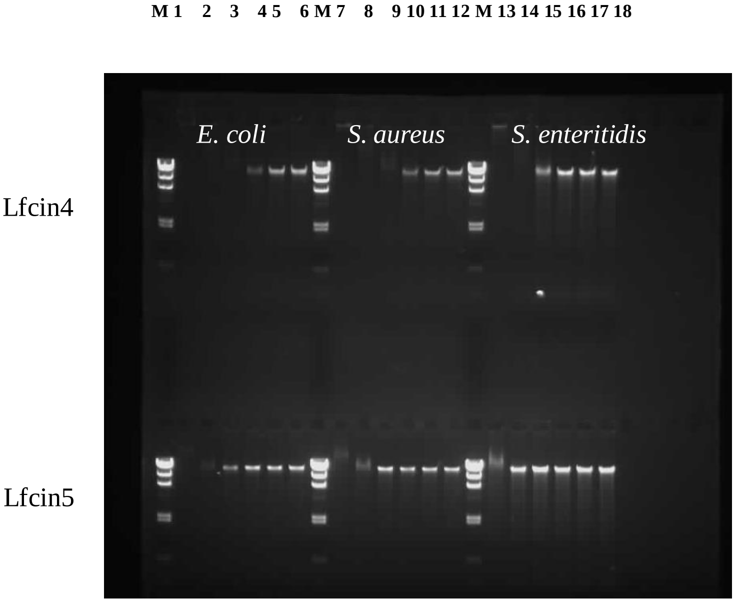


**Supplementary Figure 2 Full length gel retardation analysis of the binding of Lfcin4 and Lfcin5 to genomic DNA.** M: DNA Marker λDNA/*Hind*III. Lanes 1-6: genomic DNA from *E. coli* CICC21530; Lanes 7-12: genomic DNA from *S. aureus* ATCC25923; Lanes 13-18: genomic DNA from *S. enteritidis* CVCC3377. The mass ratios of peptides and genomic DNA were 10, 5, 2.5, 1, 0.5, 0, respectively.
